# Facilitators and barriers to community-led monitoring of health programs: Qualitative evidence from the global implementation landscape

**DOI:** 10.1371/journal.pgph.0003293

**Published:** 2024-06-20

**Authors:** Alana R. Sharp, Ngqabutho Mpofu, Elise Lankiewicz, Beatrice Ajonye, Ndivhuwo P. Rambau, Stefanie Dringus, Brian Honermann, Ngozi Erondu, Asia Russell, Kenneth Mwehonge, Cláudia Aguiar, Naïké Ledan, Matthew M. Kavanagh

**Affiliations:** 1 O’Neill Institute for National and Global Health Law, Georgetown University, Washington, District of Columbia, United States of America; 2 Treatment Action Campaign, Johannesburg, South Africa; 3 Andelson Office of Public Policy, amfAR, Washington, District of Columbia, United States of America; 4 International Community of Women Living with HIV Eastern Africa, Kampala, Uganda; 5 Global Institute for Disease Elimination, Abu Dhabi, United Arab Emirates; 6 Health GAP, Washington, District of Columbia, United States of America; 7 Coalition for Health Promotion and Social Development, Kampala, Uganda; 8 School of Health, Georgetown University, Washington, District of Columbia, United States of America; Johns Hopkins Center for Health Security: Johns Hopkins University Center for Health Security, UNITED STATES

## Abstract

Achieving the global HIV, tuberculosis, and malaria targets will require innovative strategies to deliver high quality and person-centered health services. Community-led monitoring (CLM) is a rapidly proliferating health systems strengthening intervention for improving healthcare services and documenting human rights violations, through social empowerment and political accountability. Driven in part by increasing financial support from donors, a growing number of countries are implementing CLM programs. This study aimed to identify early challenges and lessons learned from CLM implementation, with the aim of informing and improving the implementation of CLM programs and ultimately achieving greater impact on the delivery of services. Twenty-five CLM implementors representing 21 countries participated in an interview. Early generation of buy-in from diverse stakeholders was noted as critical for CLM success. Leveraging existing networks of service users and community organizations to implement CLM also helped to maximize program reach and resources. Uncertainty around CLM’s purpose and roles among CLM stakeholders resulted in challenges to community leadership and ownership of programs. Respondents also described challenges with underfunded programs, especially advocacy components, and inflexible donor funding mechanisms. Critical capacity gaps remain around advocacy and electronic data collection and use. With the rapid expansion of CLM, this study serves as an important first step in characterizing challenges and successes in the CLM landscape. Successful implementation of CLM requires prioritizing community ownership and leadership, donor commitment to sustainable and reliable funding, and strengthened support of programs across the data collection and advocacy lifecycle.

## 1. Introduction

The joint efforts of governments, donors, and civil society have achieved tremendous progress in the fight against HIV, tuberculosis, and malaria as public health threats [1–3]. Yet despite progress in many countries toward elimination and control targets, several countries and populations continue to be left behind [4].

Improving healthcare quality and access is an urgent priority in the fight against the three diseases, one requiring a multi-faceted approach to both identifying and addressing a wide range of clinical, social, and economic health-related barriers and enablers. One proposed avenue for improving service delivery is the strengthening of accountability structures within health systems through community-led monitoring.

Community leadership in identifying gaps and advocating for change is not a new practice. Since the 1970s, a variety of community-based initiatives to monitor health systems have been described, including community scorecards, citizen report cards, and health facility committees [5, 6]. Today, community-led monitoring (CLM) is commonly defined by a service user- and community-driven approach that not only identifies gaps but uses routine data collection and advocacy to apply pressure on decision-makers to improve service delivery, generate political will, and improve accountability [7]. As such, CLM is typically implemented as a routine cycle of information gathering (at the community or facility level), analysis of data to identify gaps and barriers, development of solutions to issues identified in the data, feedback of findings and solutions to stakeholders, and advocacy for changes to policy and practice.

This central focus on advocacy and community ownership also differentiates the CLM model from traditional monitoring and evaluation techniques. While the accuracy of community data is important, the CLM model intentionally prioritizes context-specific knowledge, participatory methodologies, and local decision-making over commonly held standards for monitoring and evaluation (M&E). Indicators are developed based on the expertise and perspectives of the communities most familiar with the challenges in the healthcare system, and often capture aspects of patient centered healthcare like understanding why service users are lost to follow up and documenting human rights violations. This approach prioritizes responsiveness and adaptability, allowing communities to define their own monitoring indicators and methodologies. Additionally, CLM programs are typically operated out of small, local civil society organizations with support from international donors, and as such the breadth and depth of monitoring is typically limited by institutional capacity and funding levels.

Recent years have seen growth in CLM implementation, particularly HIV-focused programs. With increasing recognition that global HIV targets will not be achieved without innovative strategies to deliver high-quality patient-centered services, CLM has sparked donor interest and has been highlighted as relevant to pandemic preparedness efforts. Since 2020, PEPFAR has required CLM in all countries receiving PEPFAR funding [8] and the Global Fund currently supports CLM through allocation funding, Strategic Initiatives, [9] and the COVID-19 Response Mechanism (C19RM).

Despite increased awareness and donor support, many funded CLM programs have faced significant challenges to initial implementation and there exists little formalized research on CLM to date. Social accountability mechanisms like CLM remain an understudied approach to health systems strengthening, and assessments of impact on services delivery have been mixed in both health and development more broadly [10, 11]. These variable results have been attributed to broad definitions of social accountability and evaluations that lack nuanced consideration of the complex and highly-context dependent nature of social accountability work [12, 13]. Accordingly, understanding the potential impact of social accountability work is not just about asking if it works, but seeking to understand the conditions that facilitate success [12].

As several CLM programs conclude their first year (or more) of implementation, and with a rapid growth in CLM implementation anticipated in the near-term, a critical moment has emerged to elucidate the key facilitators and barriers to success emerging from CLM work. This study aims to identify the challenges and associated lessons learned from early implementation of CLM, with the aim of ensuring that investments in CLM implementation are likely to achieve impact and improvements in the quality of care.

## 2. Materials and methods

### 2.1 Data collection

Participants were recruited through a two-stage process. First, respondents participated in a brief screening survey that gathered informed consent and data on key parameters of the participants’ CLM programs. Participants consented simultaneously to the screener and interview process. The screening survey was distributed in five ways: 1) directly to CLM programs identified by the authors or funders; 2) via research and advocacy networks; 3) via social media; 4) on electronic notice boards of global public health institutions and universities; and 5) through snowball sampling in which respondents were encouraged to share contact information of other individuals and/or programs who may be interested in participating in the project and were able to forward the link to the screening questionnaire. The screening questionnaire was administered through Qualtrics and was available in English, French, Portuguese, Russian, and Spanish (S1 Text).

The screening process was designed to limit the sample and findings specifically to CLM programs, while excluding other community systems strengthening interventions and classic M&E programs. Participants that self-reported being part of a CLM program and met two out of three inclusion criteria were included in the study: 1) implementation is led by a local civil society organization, key, vulnerable, or priority populations, or people living with or impacted by HIV, tuberculosis, or malaria; 2) activities include collecting data on healthcare quality and access; and 3) activities include advocating for solutions and working with decision-makers for change. In addition, participants from programs operating with a regional or global focus (i.e. not implementing in one country) were excluded.

Participants who met the inclusion criteria were invited to participate in an in-depth interview. In cases where multiple respondents from the same CLM program responded (based on the program name) only the first respondent to complete the survey was invited to participate in an interview. In-depth interviews guides were semi-structured and explored key aspects of their program, with a particular focus on challenges, successes, key learnings, and recommendations for best practices (S2 Text).

Interview guides were developed through consultations with CLM implementors and contained five key sections: governance and structure; financing; data collection, analysis, and reporting; advocacy; and engagement with external stakeholders. Interviews were conducted on recorded Zoom calls in the respondent’s preferred language and translated to English and transcribed verbatim. Interviews were conducted by two researchers outside of the community, with positionality shaped by their expertise in public health research and evaluation.

**Fig 1:**
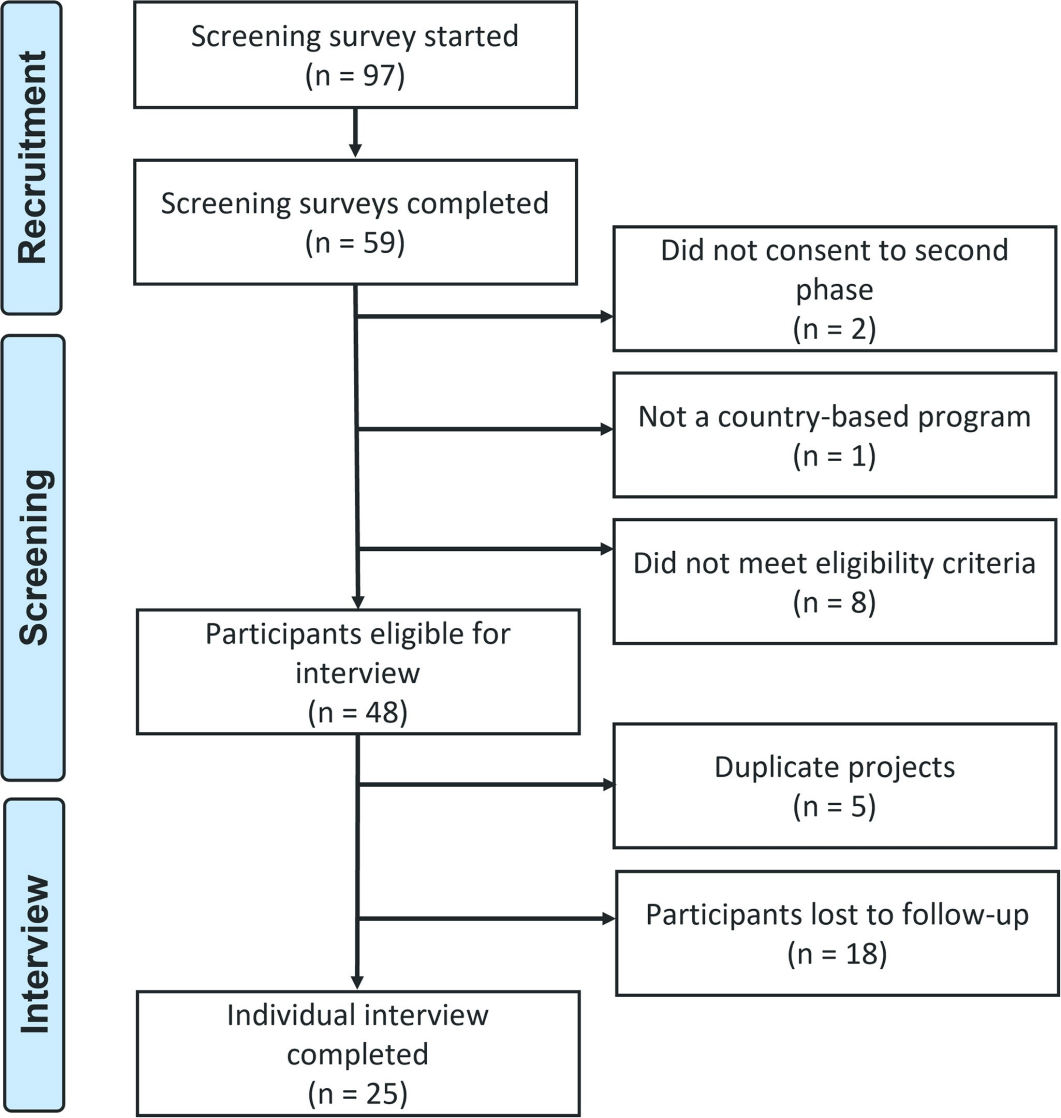
Participant recruitment flowchart.

An initial 97 respondents began the screening survey, of whom 59 completed the entire survey and 48 were eligible to participate in an interview and 25 completed an interview (Fig 1). Of these, 25 (100%) self-identified as being involved in a CLM program or other initiative to use monitoring data to advocate for better healthcare services (Table 1). Among the final sample that participated in the interviews, 18 (72%) fulfilled all three eligibility criteria; four (16%) were part of programs not led by a local civil society organization, key populations, and/or people living with affected by the three diseases; two (8%) were part of programs without advocacy activities; and one (4%) did not report collecting health facility data.

**Table 1:** Participant characteristics (N = 25).

Characteristic	Total	N	%
Currently working on a CLM program*	25		
Yes		25	100%
No		0	0%
Respondent’s position in the CLM program	25		
Staff at an organization involved in implementing the monitoring project		17	68%
Community member involved in the project		11	44%
Other		8	32%
Advisor / consultant / technical assistance provider		6	24%
International donor		1	4%
Government / Ministry of Health		0	0%
UN Organization		0	0%
Focus of the CLM program	25		
HIV/AIDS		23	92%
Tuberculosis		18	72%
Human rights		17	68%
COVID-19		14	56%
Malaria		7	28%
Other		5	20%
Geographic region of CLM program	25		
Western Africa		8	32%
Eastern Africa		7	28%
Middle Africa		3	12%
Central Asia		2	8%
Caribbean		1	4%
Eastern Europe		1	4%
South-eastern Asia		1	4%
Southern Africa		1	4%
Southern Asia		1	4%
Types of activities performed by CLM program	25		
Collecting data on healthcare quality and access at facility and/or community level*		24	96%
Developing an advocacy strategy to resolve issues identified in the data		23	92%
Advocating for solutions and working with decision-makers to implement change*		23	92%
Disseminating the data to key stakeholders		22	88%
Identifying service-related needs and deficits impacting the community		21	84%
Analyzing and interpreting data to find solutions and key action points		20	80%
Monitoring changes over time, looking for trends and impact		19	76%
Other		2	8%
None of the above		0	0%
Who is leading CLM implementation*	25		
Local civil society organizations		17	68%
Key, vulnerable, or priority populations		12	48%
People living with, and communities impacted by HIV, malaria, or TB		12	48%
Nonprofit healthcare organization		9	36%
Other		5	20%
The program donor (for example, the Global Fund or PEPFAR)		3	12%
Ministry of Health or other government body		2	8%
International civil society organizations		2	8%
A university or academic institution		2	8%
Not yet determined		0	0%
Who is funding the CLM program	25		
The Global Fund to Fight AIDS, Tuberculosis and Malaria		13	52%
U.S. government (PEPFAR, CDC, USAID)		9	36%
Other		6	24%
U.N. Organization (UNAIDS, UNDP, etc)		4	16%
Stop TB Partnership		1	4%
Private foundation or other donor		1	4%
Self-funded		1	4%
Roll Back Malaria		0	0%
Country government / Ministry of Health		0	0%
The project is still in the planning stages and no funding has been acquired		0	0%

* Indicates question used in exclusion criteria

### 2.2 Data analysis

Deductive hierarchical coding was used with a multi-level codebook developed from the interview guide. Each interview transcript was reviewed separately by two researchers. Discrepancies within coding were resolved via discussion with the broader research group. The preliminary codebook was iteratively refined through this group coding approach until consensus was reached. Purposeful thematic analysis of the coded data was then conducted, guided by the approach of Nowell et. al. [14]. Thematic analysis relied heavily on researcher triangulation, with researchers discussing identified themes until consensus was reached to support analytic validity [14, 15].

### 2.3 Ethical approval

This study was approved by the Georgetown University institutional review board. Electronic written informed consent was obtained from all participants as part of the Qualtrics screening survey. All data were collected between January and March 2022.

## 3. Results

### 3.1 Participants

Twenty-five respondents participated in individual interviews. Participants were from 21 countries, with representatives primarily from Western Africa, Eastern Africa, and Middle Africa (Table 1). Respondents were primarily staff working for an organization implementing the CLM program and/or community members involved in the project. Implements from CLM projects focusing on HIV/AIDS, tuberculosis, malaria, COVID-19, and human rights were included in the sample.

### 3.2 Themes

Thematic analysis revealed implementers’ perceptions of common facilitators and barriers to successful CLM implementation.

#### 3.2.1 Facilitators to CLM implementation

*Negotiating stakeholder relationships*. Part of maintaining successful relationships with stakeholders frequently required framing CLM as a collaboration between community, funders, and government. This positioning was achieved in several ways. First, many programs noted they had to engage a diversity of stakeholders early, both to explain the CLM model and to convey the program as a non-oppositional partnership.

*I think the greatest thing that we did was to acquire high political will*, *which we did through the observation*, *and the acknowledgement and utilization of the current status quo…we have the [Ministry of Health] which is the clinical partner with regards to HIV*, *TB and malaria; and then*, *we’ve got—from a strategic viewpoint—the National AIDS Council*. *So*, *we looked at those entities*, *and we said*, *what role should they play in CLM? It was also important for us to set-up an entity that will not be viewed as challenging the government*. *Let’s come together and*, *from the outset*, *create a platform together*.

*(respondent from Eastern Africa)*

Secondly, respondents described the need to present data in a nuanced way, highlighting successes alongside gaps and ensuring that the program brought community-generated solutions alongside the issues they identified.

*The other thing is the way you are conducting advocacy*, *it’s like you are not coming to show their mistakes*, *their wrongs*, *you are going there to offer more opportunities*, *you are going there to offer solutions*. *If you are going there with that angle they can be able to be more open and listen to those kinds of findings or feedback*.

*(respondent from Eastern Africa)*

However, in some cases stakeholders were unconvinced of the collaborative nature of CLM and instead perceived it as an antagonistic civil society tactic. This conception was described as creating challenges with soliciting support from stakeholders and building relationships with clinic staff.

*Building on existing community structures*. Respondents repeatedly described the value in incorporating community members and key populations into CLM implementation. While a few respondents found it challenging to manage roles and priorities across diverse organizations, respondents overall found a multitude of benefits to leveraging existing networks of community organizations and service users when starting CLM programs. Existing networks were able to expand the reach of CLM programs in less resource-intensive ways, often because they covered a wider geographic area and already had skilled and knowledgeable staff.

*There’s a network of people living with HIV and AIDS*, *people who use drugs also have their own network […] commercial female sex workers also have their own groups […] then the same thing with the LGBT communities*. *If we want to collect more data also*, *we can also leverage [these networks] because with a limited amount of resources and power*, *we can’t reach everybody*. *But if we can also collect data via this network*, *it has a wider reach*.

*(respondent from Western Africa)*

In addition to the benefits for data collection purposes, these networks also served as critical pathways for disseminating CLM data back to communities, which was perceived to be a challenging but essential component of CLM.

Employing health service users and representatives of key populations as data collectors and advocates was also noted as essential for legitimizing the programs. Staffing from the community conferred several benefits, including legitimizing the program in the eye of stakeholders, service recipients feeling more at ease discussing challenges during data collection, and the opportunity to draw on firsthand expertise around community needs during data analysis and advocacy. It was also noted that while programs benefited from community staff, the monitors themselves benefited by building a knowledge base about the issues directly impacting them and developing transferable professional skills.

*But also*, *the use of the data collectors who were*, *you know*, *people from the communities—people from the districts where the study was taking place—was also an awesome thing because it empowered people to understand what it is when they are talking about COVID*, *when they’re talking about TB or HIV*. *And also*, *you would see that the capacity that was built also was that people were able to understand exactly how important data is when you are making programs*.

*(respondent from Eastern Africa)*

#### 3.2.2 Barriers to CLM implementation

*Implementer relationships with stakeholders*. A core tenet of the CLM model is programmatic leadership by civil society organizations and community members. Respondents commonly reported that funders, financial pass-throughs, and governments were leading aspects of implementation that respondents identified as rightfully belonging to the community. For one program, this meant that community members were hired as data collectors with no other participation or leadership in CLM implementation:

*So*, *the donors have the full control over how they want everything done if they’re funding them*, *and it prevents them…as the organization—to share those results with who they want to share those results…And then after they collect the data*, *the donors just take the data off their hands and do whatever they want with it*.

*(respondent from Central Asia)*

Similarly at odds with community leadership, donors and financial pass-through organizations also made significant decisions about the populations and topics to be prioritized in monitoring, as well as decisions about how community data could be shared. This included blocking the use of a publicly available data dashboard for one program.

*The big issue for [us] now is to use the dashboard to publish all the data we have*. *We still have the dashboard*, *[but] we need to have the conversation we PEPFAR […] they really don’t accept us to publish it for anyone to see*. *[…] So*, *if we can address that situation [with] OGAC and PEPFAR [to clarify that] the CLM have the rights to publish the data on their dashboard*.

*(respondent from Caribbean)*

At times, these challenges to community ownership were driven by misunderstandings about the CLM model. Funders and governments were described as unaware of CLM’s objectives, methodology, and their role in implementation. These misunderstandings made it difficult to solicit political buy-in. Additionally, some stakeholders urged CLM programs to adopt standardized, international monitoring indicators, which respondents noted led to survey instruments that were not appropriate for their local context. Where all stakeholders were able to reach consensus around the purpose of CLM early and communities obtained buy-in from the national government, this partnership facilitated engagement with local government and facilities during program implementation. Maintaining these relationships required ongoing communication, regular dissemination of results to stakeholders, and participation in meetings and events.

*Improperly-financed and underfinanced for scope of work*. Respondents consistently reported challenges funding CLM programs, both due to underfunding and delays in receiving funds from donors. A commonly-reported gap was adequately paying the ‘community monitors,’ a term referring to CLM frontline staff who collect data in the community or at health facilities. Multiple respondents reported that community monitors needed to pay out-of-pocket for costs related to data collection.

Other common funding gaps were reimbursing transportation costs during data collection, purchasing tablets for electronic data collection, and budgeting sufficiently for advocacy activities. Data collection costs were noted to take priority in budgets, leaving limited resources for advocacy staff, deliverables, and actual engagement with service providers, government, and funders.

One consequence of low salaries was high turnover of community monitors, which exacerbated financial challenges by requiring programs to invest in frequent re-training of staff.

*One of the key areas that we currently are not sure of is [*…*] are the [community monitors] going to stay long? Are we going to incentivize them enough to stay long and do this work after having invested so much in them? [*…*] Probably the answer is not yes*, *it’s a no*, *because these are people who can take their skills and capacities somewhere else*.

*(respondent from Eastern Africa)*

Low budgets were described as a consequence of donor-driven requirements, including overall CLM budget ceilings, caps on reimbursing community workers, and declining to pay for electronic data collection tools and advocacy program components. Additional challenges included opacity around funding mechanisms, lack of visibility on available funding, and challenges engaging in budget negotiations.

In addition to the amount of funding available for implementation, the funding disbursement mechanisms themselves were described as a challenge. Many respondents described not receiving indirect costs and overhead in CLM grants, which were perceived as necessary to implementing quality programming. This lack of indirect costs was especially challenging when significant staff capacity was directed toward CLM implementation. In some cases, respondents described donor funding being routed through multiple financial conduits, each deducting indirect costs at the expense of the overall programmatic budget.

The financial intermediaries created additional challenges that extended beyond funding. One report described a financial conduit refusing to release funds.

*There was an HIV program manager [with the Principal Recipient who would tell us] that no*, *we have no money for [community] observers*, *so instead of three you will continue with a single observer*. *We said “Ah*, *well*, *there is no money? How?” And I was forced to write to the Global Fund […] and the Global Fund was forced to balance the approved budget for the country and under the watchdog line*, *there was money*.

*(respondent from Middle Africa)*

In other cases, financial intermediaries created conflicts of interest, particularly in scenarios where the principal recipient of funding had authority over the programs being monitored.

Respondents suggested that direct funding of the civil society organizations leading CLM implementation, or funding passing through a non-implementing institution like UNAIDS, could mitigate these conflicts of interest. More direct funding of community organizations was additionally described as helping to build organizational capacity of community organizations.

*Establishing CLM as distinct from monitoring and evaluation*. Another commonly identified challenge was stakeholders conflating CLM with academic research and M&E. Respondents described facing questions about the validity of CLM data, with stakeholders holding CLM programs to technical research standards for sample sizes, sampling, and generalizability, often using this critique to discredit CLM findings and recommendations. However, many of the ways in which CLM can be distinguished from academic research were seen as positives for community members, particularly the timeliness of CLM data and the way in which data is truly reflective of community priorities.

*[CLM] creates a world apart from the previous world where community-led monitoring was not available*, *where we had to rely on surveys*, *where we had to rely on a situational analysis and all sorts of things that are very…what I can call very academic*, *very structured in nature*. *It’s community-led monitoring*, *where it is functional*, *where the information is available*, *and it’s stored and packaged in a manner that speaks to what communities want*, *creates an opportunity for data that is available*, *that can be shared at any moment*.

*(respondent from Eastern Africa)*

Programs also collected qualitative data that were described as being compelling, but respondents were unsure of how to present the data in a way that would be acceptable to duty bearers.

*If you pick up qualitative issues […] some of them are critically important for one site*, *but they are not for another site*. *But I think people or stakeholders*, *and even key advocacy players*, *are interested in things that affect the majority*. *So how do you transmit that information about the minority so that it makes sense?*

*(respondent from Eastern Africa)*

*Capacity gaps remain*. The routine cycle of data collection proved to be a significant challenge for multiple programs. This included navigating to remote and sometimes dangerous facilities, keeping data collection tools relevant to the priorities of the community, and figuring out affordable and usable electronic data collection systems. Where data were successfully collected, several programs reported being unable to analyze data and propose evidence-informed solutions quickly enough to be timely for advocacy. This was especially true for programs utilizing paper data collection rather than electronic data collection, which proved to demand inordinate staff effort to successfully manage and analyze.

*The data analysis takes a lot of time if you want to do it the right way…usually this is what happens—they collect data*, *then for another half a year*, *they’re working on the report*. *And then the results that are being represented are retroactive*, *right? So they’re not representing what’s currently happening*.

*(respondent from Eastern Europe)*

Respondents also noted that there were critical skills gaps among the community monitors. Despite program management staff receiving technical assistance to build capacity around data skills, community monitors often lacked those same opportunities. Providing more professional development opportunities and training for community monitors was perceived as an opportunity to both incentivize staff to stay engaged with the program and to improve the quality of data collection and direct advocacy.

*[Community monitors] are the ones at the front*. *It’s different from the generals who are in the offices*. *They are theorists where they know war*, *in theory*. *But [without capacitation] those who face the enemy*, *the target…they are not armed*.

*(respondent from Western Africa)*

Conducting activities for the advocacy phase was also identified as a challenge, with programs lacking technical advocacy skills and the human resources to allocate to advocacy. CLM programs reported successful advocacy required negotiating challenging political contexts and navigating a complexity of stakeholders responsible for different aspects of service delivery. Challenges included identifying the players capable of addressing specific issues captured by CLM data and developing tailored advocacy the strategies that would effectively reach different levels of government.

*And the other thing is there are different stakeholders that play different roles at the facility*, *so sometimes it will be an issue to do with those that have worked with construction*. *Yet for us*, *maybe we are trying to approach the Department of HIV and AIDS*, *it’s not affected by that*.

*(respondent from Central Asia)*

Many programs also found that despite sufficient technical assistance (TA) around data collection, TA for advocacy skills was nearly nonexistent. Some programs suggested that more opportunities to learn from CLM programs elsewhere might help to fill advocacy-related capacity gaps.

## 4. Discussion

The community-led monitoring model is defined by two key characteristics: first, by its emphasis on community ownership of the full cycle of activities, and secondly, that data collection must be followed by evidence-informed advocacy. This study provides an early look into the real-world experiences and challenges facing community implementers.

The reported value of community ownership, collaborative approaches, and using community data for advocacy align with previous evidence [5]. Indeed, the existing evidence base on social accountability for health services has suggested that such strategies often work through ‘soft pressure’ in which change occurs through positively shifting relationships between community and duty bearers, [16] aligning with the emphasis respondents in this study placed on generating collective buy-in and collaboration. The critical role that allies both within health systems and within civil society can play in further elevating or supporting accountability demands has been well established, further supporting the role that broad stakeholder buy-in can play in successful CLM implementation [12, 17]. Ensuring stakeholders understand the concept and importance of CLM can help to strengthen these ally-ships.

In addition to allies within health systems or donors, respondents here also cited the importance of engaging a broad coalition of civil society in CLM efforts, especially in order to build the reach of CLM efforts in a resource efficient way. The literature suggests that beyond this particular benefit, social accountability efforts that consist of or engage with a broad variety of community and civil society organizations may help to grow the social capital of the effort and help to increase bargaining power with duty bearers [18]. Interestingly, the need for CLM to representative of broad swaths of community also emerged in this study as it relates to approaches to data collection. Respondents noted that qualitative data was often dismissed by decision makers. The aggregation of voice has been proposed as an important moderator of social accountability success, aligning with challenges CLM projects faced in make the stories of individuals pressing for decision-makers [12].

The importance of hiring impacted communities to serve as data collectors highlighted by respondents in this work aligns with previous literature that suggests decision-makers perception of the legitimacy of citizen groups may play an important role in responsiveness to social accountability [16]. Respondents here highlighted that this also helps service users to build capacity and advocacy skills. Indeed, empowerment of community is a key outcome tied to social accountability in development broadly, though the potential for empowerment may be limited where state structures and processes over dictate the structure of accountability interventions [10].

In this study, participants noted that they were at time challenged by gaps in skills related to both data collection and advocacy. The inclusion of mechanisms that aimed to build capacity for collective action has been noted as an important facilitator of accountability and transparency work that engages community [18]. Respondents in this study made clear that this kind of capacity building is often under-funded within their projects.

While existing literature supports that social accountability work can be limited by challenges related to under-funding, more specific concerns related to funding structure emerged from this study [19]. Donors appear ill-adapted to financing large-scale programs that are led by community organizations, with clear barriers emerging around adequately funding core program costs. Further, traditional funding mechanisms are challenging for community organizations with limited financial capacity, with low budgets or delays in disbursements having knock-on effects on program’s abilities to implement programs and retain staff.

These findings have implications for funders, stakeholders, and CLM implementers. Funder policy is needed that emphasizes community ownership of all aspects of CLM and funders can also actively support CLM implementors when other stakeholders are challenging those roles. Such policy should outline roles and limitations of financial passthroughs and technical assistance providers. Where needed, funders may also have to facilitate preliminary meetings between government and CLM programs to build understanding of the CLM model, where community members cannot safely or effectively do so.

As the evidence base on effective CLM grows, grant allocations and structures must grow to accommodate these components like funding for community monitor pay or salary, aspects of CLM advocacy like dedicated staff and technical assistance, and the tools necessary for electronic data collection. Where program implementation is yet to begin, funders should structure requests for applications that facilitate CLM implementation by networks of community organizations to amplify reach and impact of programs.

Existing literature has emphasized the potential of social accountability efforts to improve service provider and service user relationships, quality of care, and healthcare outcomes, [10, 16, 19, 20] and despite the nascency of many of the CLM programs described here, many respondents viewed CLM as a valuable tool for elevating community voice in service delivery where implementers successfully create strong working relationships with stakeholders, service users, and civil society networks. Stakeholders have the opportunity to facilitate the implementation of high quality CLM by addressing the need for flexible, adaptive funding mechanisms and greater consensus to be built around CLM models and roles.

## 5. Conclusions

Community-led monitoring may play an important role in improving healthcare systems through community and service user empowerment. However, understanding the moderators of successful CLM implementation is critical to improving implementation moving forward. Ensuring impactful CLM implementation will require a concerted effort by international donors, governments, and other stakeholders to financially and programmatically support community organizations with the implementation of large-scale, technically complicated programs.

## Supporting information

S1 Checklist Inclusivity in global research. (DOCX)

S1 Text Brief screening tool. (DOCX)

S2 Text Interview question guide. (DOCX)

## References

[1] Joint United Nations Programme on HIV/AIDS (UNAIDS). The path that ends AIDS: 2023 UNAIDS Global AIDS Update. 2023. https://thepath.unaids.org/wp-content/themes/unaids2023/assets/files/2023_report.pdf.

[2] World Health Organization (WHO). World malaria report 2023. 2023. https://www.who.int/teams/global-malaria-programme/reports/world-malaria-report-2023.

[3] World Health Organization (WHO). Global tuberculosis report 2023. 7 Nov 2023. https://www.who.int/teams/global-tuberculosis-programme/tb-reports.

[4] World Health Organization (WHO). State of inequality: HIV, tuberculosis and malaria. 9 Dec 2021. https://www.who.int/data/inequality-monitor/publications/report_2021_hiv_tb_malaria.

[5] Baptise S, Manouan A, Garcia P, Etya’ale H, Swan T, Jallow W. Community-led monitoring: when community data drives implementation strategies. Curr HIV-AIDS Rep. 2020;17:415–21. doi: 10.1007/s11904-020-00521-2 32734363 PMC7497354

[6] Hemmings J, Wilkinson J. What is a public health observatory? J Epidemiol Commun H. 2003;57:324–6. doi: 10.1136/jech.57.5.324 12700213 PMC1732445

[7] Joint United Nations Programme on HIV/AIDS (UNAIDS). Establishing community-led monitoring of HIV services—Principles and process. 25 February 2021. https://www.unaids.org/en/resources/documents/2021/establishing-community-led-monitoring-hiv-services.

[8] PEPFAR. PEPFAR 2020 Country Operational Plan guidance for all PEPFAR countries. 2020. https://www.state.gov/wp-content/uploads/2020/01/COP20-Guidance.pdf.

[9] The Global Fund to Fight AIDS, Tuberculosis and Malaria. 2020–2022 Strategic Initiatives. 2020. https://www.theglobalfund.org/media/9228/fundingmodel_2020-2022strategicinitiatives_list_en.pdf?u=637166002510000000.

[10] Brinkerhoff DW, Wetterberg A. Gauging the Effects of Social Accountability on Services, Governance, and Citizen Empowerment. Public Admin Rev. 2015 Jun 10;76(2):274–86.

[11] Joshi A. Do They Work? Assessing the Impact of Transparency and Accountability Initiatives in Service Delivery. Dev Policy Rev. 2013;31: s29–48.

[12] Fox JA. Social Accountability: What Does the Evidence Really Say? World Dev. 2015 Aug;72:346–61.

[13] Joshi A, Houtzager PP. Widgets or Watchdogs? Conceptual explorations in social accountability. Public Manag Rev. 2012;14(2):145–62.

[14] Nowell LS, Norris JM, White DE, Moules NJ. Thematic analysis: striving to meet the trustworthiness criteria. Int J Qual Meth. 2017;16:1–13.

[15] Creswell JW, Miller DL. Determining validity in qualitative inquiry. Theor Pract. 2000;39(3):124–30.

[16] Lodenstein E, Dieleman M, Gerretsen B, Broerse JE. Health provider responsiveness to social accountability initiatives in low- and middle-income countries: a realist review. Health Policy Plann. 2017;32(1):125–140. doi: 10.1093/heapol/czw089 27375128

[17] Mcloughlin C, Batley R. The Politics of What Works in Service Delivery: An Evidence-Based Review. SSRN Electronic Journal. 2012; doi: 10.2139/ssrn.2141852

[18] Waddington H, Sonnenfeld A, Finetti J, Gaarder M, John D, Stevenson J. Does incorporating participation and accountability improve development outcomes? Meta-analysis and framework synthesis. 3ie Systematic Rev 2019;43. https://www.3ieimpact.org/evidence-hub/publications/systematic-reviews/does-incorporating-participation-and-accountability#

[19] Danhoundo G, Nasiri K, Wiktorowicz ME. Improving social accountability processes in the health sector in sub-Saharan Africa: a systematic review. BMC Public Health. 2018;18(497). doi: 10.1186/s12889-018-5407-8 29653531 PMC5899409

[20] Molina E, Carella L, Pacheco A, Cruces G, Gasparini L. Community monitoring interventions to curb corruption and increase access and quality of service delivery in low- and middle-income countries: a systematic review. Campbell Sys Rev. 2016;12(1):1–204.

