# Supplementary material for: Facilitators and barriers to community-led monitoring of health programs: Qualitative evidence from the global implementation landscape
Source: PLOS Glob Public Health. 2024 Jun 20;4(6):e0003293. doi: 10.1371/journal.pgph.0003293 (PMC11189247; doi:10.1371/journal.pgph.0003293)
Supplement: S1 Text — (DOCX) [file pgph.0003293.s002.docx]

**Best Practices Tool.**

**BRIEF SCREENING TOOL**

| **1. When was this survey filled out?** |
| --- |
| **2. Please read carefully the document describing the purpose of the study and your rights as a participant in the project. *Please indicate whether you have read the document and agree to participate in the study*.**  **What is the objective of this project?** The Global Fund to Fight AIDS, Tuberculosis and Malaria has commissioned the CLAW Consortium to evaluate the best practices for programs monitoring HIV, Tuberculosis, and Malaria healthcare service delivery. The findings from this work will be used to guide new and existing healthcare monitoring projects in implementing projects that are impactful, effective, and equitable.  **Who is eligible to participate?** Information will be gathered from existing healthcare monitoring projects. In the initial phase, any organization that self-identifies as participating in a healthcare monitoring project will be invited to participate. Programs with a focus on HIV, tuberculosis, malaria, COVID-19, or human rights will all be eligible to participate. In later phases, a subset of individuals and programs will be selected for a follow-up information gathering.  **How will participants be recruited?** Participants will be contacted by email, either by members of the CLAW consortium, or by CLAW partner organizations, or by other individuals that have participated in the initial survey. Participants may also find the initial survey through listservs or social media.  **If I agree to participate, what will I need to do?** In the first phase, participation will involve filling in an online short survey. This survey should take no longer than 10 minutes to complete. While filling in the survey, participants will be asked to indicate if they are interested in being contacted for the second phase. If requested to participate in the second phase of data collection, representatives of each project will be asked to complete a longer online quantitative survey, which will take no longer than 30 minutes. Participants may also be asked to be interviewed or to participate in a focus group to discuss their experiences running or participating in a healthcare monitoring program. All information gathering will happen virtually, although participants may choose to hold in-person meetings with colleagues in order to better respond to the questions. Participants may decide to stop participating at any phase of the project.  **What will happen with the information I share?** Information shared during all phases of the project will be used to develop a ‘best practices’ tool for the Global Fund. Participants’ names will never be made public (i.e. the findings will be de-identified). However, information may be shared at a country, regional, or disease level. All information will be stored in password-protected systems and survey findings will be stored separately from any personally identifiable information. Audio recordings during interviews and/or focus groups will be strictly voluntary, and any audio files recorded during the study will be destroyed after the text has been transcribed.  **Are there any potential risks to participating?** If you decide to participate, you may need to spend up to a total of two hours participating in data collection activities. Although every effort will be made to de-identify information that may present a reputational risk to the organization, there is the possibility that someone may be able to identify which organization or country is associated with a quote or data point.  **Are there any potential benefits to participating?** The purpose of this study is to develop data to help healthcare monitoring projects in implementing projects that are equitable and impactful. These findings may be beneficial to you, your project, and other monitoring projects in other countries.  **Can I see the results of the project?** Everyone who participates in the initial survey will receive a summary of the findings at the end of the project, by email. All participants will also be invited to participate in dissemination events, such as webinars. Finally, the Global Fund may disseminate the findings through its own networks.  **What are my rights as a participant?** As a participant, you have the right to:   - Decide if you would like to participate in the study - Agree to participate now, but later withdraw from the study - Ask questions before you agree to participate - Stop an in -progress interview, and ask not to continue - Withdraw from the study at any time - See the findings from the results of this project, even if you decide to withdraw at a later stage   **What if I have questions?** If you have any questions, please email ars379@georgetown.edu.  **____** Yes, I have read the document and *I agree* to participate in the study  ____ No, I *do not agree* to participate in the study |
| *If “No”, skip to question 23.* |
| **3. What is your name?** |
| **4. What is your email address?** |
| **5. Are you currently involved in a project that does community-led monitoring, community-based monitoring, or any other initiative to use data gathered by a monitoring program to advocate for better healthcare services?**  ___ Yes ___ No |
| *If “No”, skip to question 23.* |
| **6. What is the name of your monitoring project and/or the organization that leads it?** |
| **7. Which country is this project operating in?** |
| **8. In a few words, how would you describe the type of project that you work on (for example, “community-led monitoring” or “community scorecard”)** |
| **9. What is your position within the project / in what capacity are you filling out the survey ?** (*Select all that apply*)  ____ Advisor / consultant / technical assistance provider  ____ International donor  ____ Government / Ministry of Health  ____ UN Organization  ____ Staff at an organisation involved in implementing the monitoring project  ____ Community member involved in the project  ____ Other (*please specify*): |
| **10. Who is the funder of the project?**  (*Select all that apply*)  ____ President’s Emergency Plan for AIDS Relief (PEPFAR)  ____ U.S. Centers for Disease Control and Prevention (CDC)  ____ U.S. Agency for International Development (USAID)  ____ President’s Malaria Initiative (PMI)  ____ The Global Fund to Fight AIDS, Tuberculosis and Malaria  ____ U.N. Organization (UNAIDS, UNDP, etc)  ____ Roll Back Malaria  ____ Stop TB Partnership  ____ Country government / Ministry of Health  ____ Private foundation or other donor  ____ Self-funded  ____ The project is still in the planning stages and no funding has been acquired  ____ Other (*please specify*): |
| **11. What is the status of the funding for this project?**  ____ This is the first round of funding the project has ever received  ____ This is the second (or more) round of funding the project has received  ____ The project has never received funding from a donor  ____ Other (*please specify*) : |
| **12. In which year did this project begin?** |
| **13. What is the focus of this project?** (*Select all that apply*)  ____ HIV/AIDS ____ Tuberculosis ____ Malaria  ____ COVID-19 ____ Human rights  ____ Other (*please specify*) : |
| **14. What *types of activity* does your project include?** (*Select all that apply*)  ____ Identifying service-related needs and deficits impacting the community  ____ Collecting data on healthcare quality and access at facility and/or community level  ____ Analyzing and interpreting data to find solutions and key action points  ____ Disseminating the data to key stakeholders  ____ Developing an advocacy strategy to resolve issues identified in the data  ____ Monitoring changes over time, looking for trends and impact  ____ Advocating for solutions and working together with decision-makers to implement change  ____ None of the above  ____ Other (*please specify*) : |
| **15. In your project, *who is leading* the project implementation, including decisions about planning, staffing, data collection, advocacy, and reporting?**  (*Select all that apply*)  ____ Ministry of Health or other government body  ____ The project donor (for example, the Global Fund or PEPFAR)  ____ Nonprofit healthcare organization  ____ A university or academic institution  ____ Local civil society organizations  ____ International civil society organizations  ____ People living with, and communities impacted by, HIV, malaria, or TB  ____ Key, vulnerable, or priority populations  ____ The project is still in the planning stages and the implementation arrangements are still under discussion  ____ Other (*please specify*) : |
| **16. In your project, who *collects the data* on healthcare quality and accessibility?** (*Select all that apply*)  ____ Ministry of Health or other government body  ____ The project donor (for example, the Global Fund or PEPFAR)  ____ Consultants or other technical experts  ____ Members of communities impacted by HIV, TB, and/or malaria  ____ Staff from the organization leading the project implementation  ____ A university or academic institution  ____ The project is still in the planning stages and the data collection plan is still under discussion  ____ Other (*please specify*) : |
| **17. Does your project *train the participants* in the project (including those collecting data and performing advocacy)?** (*Select all that apply*)  ____ Implementing organization/project secretariat staff are trained  ____ All data collectors are trained  ____ All advocates are trained  ____ None of the above  ____ The project is still in the planning stages and the training plan is still under discussion  ____ Other (*please specify*) : |
| **18. Does your project *pay the participants* in the project (including those collecting data and performing advocacy)?** (*Select all that apply*)  ____ Implementing organization/project secretariat staff are paid  ____ All data collectors are paid  ____ All advocates are paid  ____ None of the above  ____ The project is still in the planning stages and the budget is still under discussion  ____ Other (*please specify*) : |
| **19. Which *types of data* are collected as part of your project?**  (*Select all that apply*)  ____ Surveys collected in healthcare facilities  ____ Surveys collected in communities  ____ Focus groups and interviews conducted in healthcare facilities  ____ Focus groups and interviews conducted in communities  ____ Monitoring data shared by donors (for example, PEPFAR MER data)  ____ Monitoring data shared by Ministry of Health/government  ____ The project is still in the planning stages and the data collection plan is still under discussion  ____ Other (*please specify*) : |
| **20. In your most recent cycle of data collection, how many health facilities did you collect data from?** (*If your project has not started data collection, please estimate how many you plan to visit*)  ____ 1 - 25 facilities  ____ 26 - 50 facilities  ____ 51 - 75 facilities  ____ 76 - 100 facilities  ____ 101 - 150 facilities  ____ 151 - 200 facilities  ____ 201 - 300 facilities  ____ 301 - 400 facilities  ____ 401 - 500 facilities  ____ More than 500 facilities  ____ The project does not collect data at health facilities  ____ The project is still in the planning stages and the data collection plan is still under discussion  ____ Other (*please specify*) : |
| **21. In your project, how are the *data used* after they are collected?** (*Select all that apply*)  ____ Data are used to prepare reports for donors  ____ Data are used to prepare reports for government  ____ Data are analyzed to identify gaps and issues in healthcare service delivery and accessibility  ____ Data are used to propose solutions to improve healthcare services  ____ Data are used to develop and implement an advocacy strategy aimed at healthcare facilities  ____ Data are used to develop and implement an advocacy strategy aimed at local and national government  ____ Data are used to develop and implement an advocacy strategy aimed at international donors  ____ None of the above  ____ The project is still in the planning stages and the data use plan is still under discussion  ____ Other (*please specify*) : |
| **22. As part of this project, we will be reaching out to some survey participants to gather more in-depth information about your project’s implementation and outcomes. This second phase may include an additional survey and an invitation to participate in an individual interview or group discussion.**  ***Are you interested in your project being considered for this second phase?***  ____ Yes  ____ No |
| **23. As part of this project, we are gathering input from a variety of people involved in community-led monitoring programs. To help us reach more participants, would you be willing to provide the name, organization, and contact information of individuals who may be interested in completing this survey? These can be people who work on your project or on a different project.**  *(This step is optional and does not impact your participation in the survey. Any contact information you provide will only be used to share the survey invitation).*  Contact 1 : ____________________________________________________________________  Contact 2 : ____________________________________________________________________  Contact 3 : ____________________________________________________________________ |
| **24. *Thank you for your time and participation. If you have any questions, please reach out to ars379@georgetown.edu.*** |
