# Supplementary material for: Facilitators and barriers to community-led monitoring of health programs: Qualitative evidence from the global implementation landscape
Source: PLOS Glob Public Health. 2024 Jun 20;4(6):e0003293. doi: 10.1371/journal.pgph.0003293 (PMC11189247; doi:10.1371/journal.pgph.0003293)
Supplement: S2 Text — (DOCX) [file pgph.0003293.s003.docx]

**Best Practices Tool.**

**INTERVIEW TOOL**

GENERAL QUESTIONS : FOR ALL PARTICIPANTS

| **1. [INTERVIEWER] Please indicate the date this interview or focus group was conducted and the approximate length of the interview or focus group.**  **Date (dd/mm/yy): Length (hh/mm):** |
| --- |
| **2. [INTERVIEWER] I have shared with you a document describing the purpose of this study and your rights as a participant. *Please indicate whether you have read the document and agree to participate in the study*.**  **____** Yes, participant *has consented* to continue with the interview  ____ No, the participant *does not agree* to continue with the interview |
| *If “No”, skip to question 22.* |
| **3. What is your name?** |
| **4. What is your email address?** |
| **5. What is the name of your monitoring project and/or the organization that leads it?** |
| **6. Which country is this project operating in?** |
| **7. What is your position within the project / in what capacity are you participating in the interview or group discussion ?** (*Select all that apply*)  ____ Advisor / consultant / technical assistance provider  ____ International donor  ____ Government / Ministry of Health  ____ UN Organization  ____ Staff at an organisation involved in implementing the monitoring project  ____ Community member involved in the project  ____ Other (*please specify*): |

QUESTION BANK

| DOMAIN & OBJECTIVES | SAMPLE QUESTION |
| --- | --- |
| **SETTING UP PROGRAMS / STRUCTURE / GOVERNANCE**  **Objectives:**   1. Explore challenges during setting up / implementing programs 2. Identity implementation arrangements that produce the most impactful programs 3. Elucidate best practices in setting up and implementing CLM programs | **How did this CLM project start? Who came up with the idea and why?**  *(Probe : sectors/organizations involved)*  **How was it introduced?**  *(Probe: As community-led monitoring or community-based monitoring? An M&E project / strategy or an advocacy project / strategy? How was it introduced to communities? What has this meant for meeting the objectives of CLM?)*  **How is your project implemented?**  *(Probe : which organization(s) are leading, how does funding flow from donor(s), what are the different roles for organizations part of the project)*  **What *challenges* has your project’s set- up/implementation/governance arrangement faced?**  *(Probe : buy-in / sign-off from government, coordination between organizations, dividing funding and leadership roles, communication between governance entity and the larger CLM (e.g. district / regional coordinators, community monitors))*  **What has worked well to ensure buy-in of, and engagement with, government / health care system, communities and health system users?**  *(Probe : Staffing from across civil society, forums for community input, advisory boards, etc)*  **Have KP groups been involved? If yes, please describe in detail how? If no, why not?**  **What sorts of people / roles are needed on the CLM project team? What skill sets are needed to be able to carry out CLM?**  **How were communities / health facilities sampled or chosen?**  *(Probe: concerns about the way they were sampled? Representation of findings?)*  **Do you feel your project is able to operate in a truly independent manner?**  **What about your project’s set-up/ implementation/ governance arrangement *has worked well / what would you suggest as the elements of best practice?*** |
| **FUNDING**  **Objectives:**   1. Identify donor practices that are barriers to programs 2. Identify donor practices that enable programs 3. Elucidate best practices in budgeting and financing | **How is your project currently funded?**  *(Probe : multiple donors, how many years is it funded for? Was it clear at the start of the funding cycle that this would be a multi-year project?)*  ***Which funding stream? Example:***  *If a GF supported country* - is funding received through core country allocation or through strategic initiative?  *If a PEPFAR supported country* - is funding received through the ambassador’s small grants program or through core funding, UNAIDS?  How has that impacted implementation / sustainability / reliability of funding?  **Was there any coordination of funding streams from different donors?**  *(Probe: how do funding mechanisms differ between different funders)*  **How does your funding come to you?**  *(Probe: directly to you? Who does it flow through? Are they health providers? Do you assess their programs? Did you face any challenges with funding disbursement which would be connected to monitoring their services?)*  **What, if any, concerns/challenges do you have about your project’s current budget, funding source(s) and funding arrangements?**  *(Probe : are all desired activities fully funded, which parts are underfunded, reporting requirements, sustainability, on-time disbursement of funds, small grants passing through many recipients - if yes, who? Are they healthcare providers - specifically ones that you monitor?)*  **If you faced challenges around receiving funding on time and in full, how has this impacted implementation?**  *(Probe : delays in funding decisions, changes in PR/IPs)*  **Are there any other funding practices or requirements that act as barriers to implementing CLM? What is the impact of these issues on programme effectiveness?**  *(Probe: restrictions on the type of topics able to advocate for, timeline and volume of reporting)*  **What do / could donors do to help support successful implementation of CLM programs?**  (*Probe: models of financing; direct funding vs. through a third party; Is there any assistance to develop financial accountability within the organization?)*  **What would you suggest as best practices related to funding?** |
| **DATA COLLECTION, STORAGE AND SHARING**  **Objectives:**   1. Identify which technical arrangement works best for data collection and storage? 2. Explore what challenges projects have faced around data sharing? 3. Elucidate best practices in data collection, storage and sharing. | **How does this project collect data?**  *(Probe : Facility- vs. community-based, regularity of data collection)*  **What method(s) is your project using to collect and store data?**  *(Probe : electronic vs. paper-based, DHIS vs. CommCare, transitioning from paper-based to electronic)*  **What was the process of designing the data collection tools and setting up data collection procedures?**  *(Probe : Who led the process? Who was involved in the process? How were indicators developed? Were there standardised templates? How much flexibility? Revisions of data tools between cycles of data collection? Are data flows clearly outlined?)*  **Please describe the team’s role in designing the data collection process, tools and indicators?**  *(Probe: what is your team’s ownership?)*  **Who collects the data for your project?**  *(Probe : healthcare users, people impacted by the diseases, payment of data collectors, training and capacity to use electronic data collection tools, independence of data collectors?)*  **How often is data collected?**  *(Probe : one-off vs. regular data collection, who decides the schedule of data collection)*  **How well do the tools work to collect useful / meaningful data? What are the gaps? What could be improved?**  *(Probe: Redundancy in some indicators, useful indicators missing)*  **Is the project able to adapt / customise the tools as needed?**  *(Probe: How much should tools be predetermined / standardised vs. customisable / flexible? How much reliance on TA providers?)*  **Who analyses the data that your project collects?**  *(Probe: what lens is brought to it? Capability / confidence of the team that’s analysing data? How much capacity has been transferred to CSOs? Is there training / support? How much reliance on TA providers?)*  **How is data from the project stored?**  *(Probe: who stores it, where is it stored, who has access to it, how secure is storage)*  **Who owns the data collected in your project?**  *(Probe : servers separate from government, access by government to data, how decisions are made around publication of data)*  **How is the data from your project used?**  *(Probe: who uses the data? For what purpose?)*  **What are the greatest *challenges* your project is facing around data collection? Data storage? Data management?**  **What about your data collection and storage system/process *has worked well*?**  **What challenges, if any, has your project faced with sharing data?**  *(Probe : government/donor not permitting public data sharing, waiting for approvals / protocols, how project overcame challenges)*  **What has worked well in your project for sharing data?**  *(Probe: having a link to a digital database, community monitors having access to facility data)*  **Is data publicly available?**  *(Probe: what was the process by which they were made available, challenges faced, transparency of data assisting in carrying out work)*  **What would you suggest as best practices related to data collection, storage and sharing?** |
| **ADVOCACY**  **Objectives:**   1. Identify the prevalence of advocacy components in healthcare monitoring projects 2. Determine which advocacy strategies are the most likely to achieve impact | **How has your project used the data it collects to advocate for improvements in healthcare access and quality?**  *(Probe : solutions visits in facilities, advocacy at local, regional, national, and international levels, written reports, media briefings, articles)*  **How do you develop solutions to the gaps you’ve identified?**  *(Probe: solutions visits in facilities, which solutions that you advocated for have been the most impactful?*  **Who usually leads and implements the advocacy process?**  *(Probe: are advocates trained, are they paid, is pay enough?)*  **How are messages and strategies developed?**  *(Probe: who comes up with the messaging? What are the tools used? How do you follow up on implementation?)*  **Which duty-bearers/stakeholders does this project engage with as part of its advocacy work? How is this done?**  *(Probe : community engagement, facility visits, international donor, donor’s implementing partners, ministry of health, public/media, what forums advocacy happens in)*  **Which types of advocacy and duty-bearer engagement have been *the most effective* in achieving impact?**  *(Probe : types of asks that duty-bearers are most responsive to, types of engagement that work well)*  **Which types of advocacy and duty-bearer engagement have *faced the greatest challenges* in achieving impact?**  *(Probe : Where does the biggest push-back happen and how to achieve impact)*  **Which types of *participants and voices* have been the most helpful in achieving impact in the project’s advocacy work?**  *(Probe : service users, communities, civil society organizations, people living with the three diseases, technical experts, other allies)*  **Which types of *data* have been the most helpful in achieving impact in the project’s advocacy work?**  *(Probe : specific indicators / data points, types of surveys, qualitative vs. quantitative, community- vs. facility-based)*  **Has your project experienced any push-back from governments or donors about the credibility or generalisability of the data collected?**  *(Probe: data not being representative enough / a large enough sample?)*  **Has your project experienced any pushback on access to data collection due to advocacy activities?**  *(probe: restrained access to health facilities)*  **Is it safe for civil society organizations to advocate to government? What strategies has your project taken to address this?**  *(Probe : environments with discriminatory laws and human rights violations, contexts with significant stigma and discrimination, countries with active political violence)*  **Does your project advocate to any service providers other than government?**  **What could funders do to support more effective advocacy?**  *(Probe: providing funding for websites, supporting more media accessibility, supporting cross-country efforts)*  **What would you suggest as best practices related to advocacy?** |
| **ENGAGEMENT WITH FUNDERS & GOVERNMENTS**  **Objectives:**   1. Identify best practices to work with governments and donors 2. Explore strategies for getting government buy-in while preventing conflicts of interest | **What strategies has your project used to engage with government as part of this project?**  *(Probe : data sharing, advocacy, public forums/meetings, coordination/collaboration on project activities)*  **What strategies has your project used to engage with donors as part of this project?**  *(Probe : data sharing, advocacy, public forums/meetings, coordination/collaboration on project activities)*  **Which types of engagement with government and donors have been the most *helpful* in facilitating change and increasing accountability?**  *(Probe : large forum vs. one-on-one discussions, public partnership vs. collaboration on project)*  *What role should the national HIV response agency in the country play?*  *What do you recommend should be done to enforce CLM compliance?*  **Which types of engagement with government and donors have been the most *challenging or harmful* in facilitating change and increasing accountability?**  (Probe: have these challenges impeded the delivery of authentic CLM in any way?)  **Please explain if you have experienced an inability to address issues with government or donors due to power dynamic, intimidation or fear?**  **What steps has the project taken / could take to ensure its independence from governments and donors?**  *(Probe : conflicts of interest involved with government involvement, limiting scopes of engagement)*  **In which areas has your project been the most successful in getting government and donor buy-in/support/receptiveness? Which have faced the greatest challenges?**  *(Probe : data collection vs. advocacy, which types of demands are most often listened to/dismissed)*  **What, if any, misunderstandings or concerns does your project’s donor/government have about healthcare monitoring programs?**  *(Probe : roles and responsibilities, areas project has had to reinforce with donor/government, capability of community to carry out CLM activities)*  **Which challenges, if any, has your project faced around conflicts of interest?**  *(Probe : donor pushback around civil society organizations conducting monitoring, government/donor being involved in monitoring of government/donor-funded healthcare system, independence of CLM implemnters)*  **What would you suggest as best practices related to engagement with funders and governments?** |
| **TECHNICAL ASSISTANCE (TA)** | **What types of TA has your project received that have been the most useful?**  **Do you think that the TA has transferred skills and knowledge in a way that leads to sustainable CLM?**  **What types of TA has your project received that haven’t been particularly useful?**  **What types of TA would your project benefit receiving / more of?**  **What would you suggest as best practices related to projects receiving useful TA?** |
| **OUTCOMES**  **Objectives:**   1. **Identify where projects have achieved the greatest successes** 2. **Triangulate outcomes data with other quantitative and qualitative data to see which practices have greatest impact** | **What are the *biggest successes* your project has achieved so far?**  *(Probe : building organizational capacity, bringing numerous stakeholders together, elevated the voices of communities and people impacted by diseases, built/strengthened dialogue with duty-bearers, increased accountability, achieved outcomes in healthcare quality, increased funding for healthcare programs)*  **What areas of your project have been the *most challenging* to move forward / achieve success in? *struggled the most* in achieving successes so far?**  **What do you credit as the most important *drivers* of your project’s success?**  *(Probe : strong collaboration across civil society, well-trained staff, sustainable funding, technical assistance, high-quality data, strong advocacy effort)*  **What are some of the reasons your project has faced challenges/not achieved its outcomes?**  *(Probe : financing, human resources, donor and government issues, project governance, discriminatory laws, conflict of interests)*  **What could be done / what is needed to help support projects in achieving success?**  **What is needed to support sustained / long-term implementation of CLM programs?**  **How did your project build capacity and scale up?**  *(Probe: capacitating core group of implementers)*  **What works well to share challenges, and learnings within and between projects / countries?**  **What would you consider *“The Essential Conditions to Conduct CLM”?***  *(Probe: democratic state, legal framework, independent Civil Society etc.)* |
| **COVID-19-SPECIFIC QUESTIONS** | **How has your project been affected by COVID-19?**  *(Probe: main challenges, social mobilisation and engagement paused, diversion of funds, limited human capacity, CLM implementers taking on additional roles / responsibilities)*  **Are frontline workers paid COVID-19 risk allowance?**  **How has your project adapted to COVID-19?**  *(Probe : new questions added to surveys to capture impact of COVID-19, challenges in data collection and protection of participants, political disruption)*  **Have there been any CLM innovations that were discovered / developed as a result of COVID19?**  **What adaptations could be documented as Best Practices?** |
